# Cancer stem cells and drug resistance in cancer: molecular mechanisms and therapeutic targets

**DOI:** 10.1186/s43556-026-00516-2

**Published:** 2026-07-17

**Authors:** Hayam Hamdy, Youzhou Li, Chen Li, Zhuoxuan Li, Xin Liu, Ensong Sun, Doaa Safwat, Junling Shen, Hui Li, Jianwei Sun

**Affiliations:** 1 Yunnan Key Laboratory of Cell Metabolism and Diseases, State Key Laboratory for Conservation and Utilization of Bio-Resources in Yunnan, Center for Life Sciences, School of Life Sciences, The Affiliated Hospital of Yunnan University, Yunnan University, Kunming, China; 2 Department of Forensic Medicine and Toxicology, Faculty of Veterinary Medicine, New Valley University, New Valley, Egypt; 3 Department of Scientific Research, Yunnan Cancer Hospital, The Third Affiliated Hospital of Kunming Medical University, Peking University Cancer Hospital Yunnan, PekingKunming, China; 4 NHC Key Laboratory of Healthy Birth and Birth Defect Prevention in Western China, First People’s Hospital of Yunnan Province, Kunming, China

**Keywords:** Cancer stem cells, Drug resistance, Tumor microenvironment, Epithelial-mesenchymal transition, Immunotherapy, Metabolic reprogramming

## Abstract

Cancer therapy has advanced substantially through targeted therapies and immunotherapy; however, durable clinical responses remain limited by the development of drug resistance. Increasing evidence identifies cancer stem cells (CSCs) as central drivers of therapeutic failure, tumor recurrence, metastasis, and minimal residual disease. CSCs possess self-renewal and differentiation capacities together with remarkable adaptability under therapeutic stress, enabling long-term tumor maintenance and regeneration. CSC-mediated resistance arises through coordinated intrinsic and extrinsic mechanisms. Intrinsically, CSCs employ multiple survival programs, including cellular quiescence, enhanced DNA damage response and repair, ATP-binding cassette transporter-mediated drug efflux, apoptosis evasion, and metabolic reprogramming. Extrinsically, these mechanisms are reinforced through dynamic interactions with the tumor microenvironment (TME), particularly hypoxic and perivascular niches that support stemness and therapeutic tolerance. Importantly, CSCs are increasingly recognized as dynamic cellular states rather than fixed populations and exhibit marked plasticity through reversible transitions between stem-like and non-stem states, frequently mediated by epithelial–mesenchymal transition (EMT). This plasticity promotes intratumoral heterogeneity and replenishes resistant cell populations. In this review, we provide a comprehensive synthesis of the molecular and microenvironmental mechanisms underlying CSC-driven drug resistance and critically discuss emerging therapeutic strategies targeting CSC plasticity, niche interactions, metabolic adaptation, and immune evasion. Collectively, these insights support the development of integrated multi-target therapeutic approaches to improve long-term clinical outcomes.

## Introduction

Cancer remains one of the leading causes of morbidity and mortality worldwide despite substantial advances in early detection and treatment. Over recent decades, cancer management has evolved from conventional approaches including surgery, radiotherapy, and cytotoxic chemotherapy toward more precise therapeutic modalities such as targeted therapy and immunotherapy [1] These advances, supported by deeper insights into oncogenic signaling and tumor genomics, have improved clinical outcomes across several malignancies [2]. Nevertheless, durable therapeutic benefit remains limited by the emergence of drug resistance, which continues to drive treatment failure, disease recurrence, and tumor progression [2, 3].

Traditionally, therapeutic resistance has been attributed to genetic alterations, intratumoral heterogeneity, and activation of compensatory signaling pathways [3]. Although these mechanisms are well established, they do not fully explain persistent tumor survival following treatment. Growing evidence identifies cancer stem cells (CSCs) as critical contributors to resistance. CSCs represent a distinct tumor subpopulation characterized by self-renewal capacity, differentiation potential, and remarkable adaptability under therapeutic stress [4–7]. These cells display intrinsic resistance to chemotherapy, radiotherapy, targeted therapies, and immunotherapy [8, 9], and are increasingly recognized as key drivers of minimal residual disease, metastasis, and tumor relapse [10].

The molecular basis of CSC-mediated resistance involves coordinated interactions between intrinsic and extrinsic mechanisms rather than reliance on a single pathway. Intrinsically, CSCs activate multiple survival programs, including quiescence, enhanced DNA damage response (DDR), increased DNA repair capacity, ATP-binding cassette (ABC) transporter activity, resistance to apoptosis, and metabolic reprogramming [11, 12]. Among these mechanisms, DDR serves as a major protective axis that enables CSCs to detect, repair, and tolerate genotoxic stress while preserving genomic integrity during therapeutic exposure [13, 14]. Extrinsically, these cell-intrinsic programs are reinforced through dynamic interactions with the tumor microenvironment (TME), where specialized niches—particularly hypoxic and perivascular regions- provide biochemical and structural support for CSC maintenance and survival [9].

Current understanding of CSC biology has also shifted from a rigid hierarchical model toward a dynamic framework in which cellular plasticity plays a central role. Non-stem tumor cells may reacquire stem-like characteristics through dedifferentiation processes, particularly via epithelial–mesenchymal transition (EMT), while CSCs themselves can transition into a more differentiated but highly adaptable state [15, 16]. Emerging evidence further implicates immune evasion and metabolic flexibility as key contributors to CSC survival across diverse therapeutic contexts [17, 18].

Despite increasing recognition of their biological importance, effective eradication of CSCs remains challenging. Existing therapeutic approaches frequently fail to eliminate these resistant populations, allowing tumor regeneration and continued disease progression. Therefore, a more comprehensive understanding of the molecular and cellular mechanisms governing CSC-mediated resistance is essential for developing more durable and effective therapeutic strategies.

In this review, we provide a comprehensive and critical overview of current knowledge regarding CSC-driven drug resistance. We first discuss the defining biological characteristics of CSCs associated with therapeutic resistance, followed by an examination of the major molecular mechanisms involved, including DNA repair, drug efflux, apoptosis resistance, metabolic adaptation, and key signaling pathways. We then explore the contribution of the TME and CSC niches, together with the roles of cellular plasticity and EMT. Finally, we summarize current and emerging therapeutic approaches targeting CSCs and outline future directions for improving clinical outcomes.

## Molecular mechanisms underlying CSC-mediated drug resistance

**Fig. 1:**
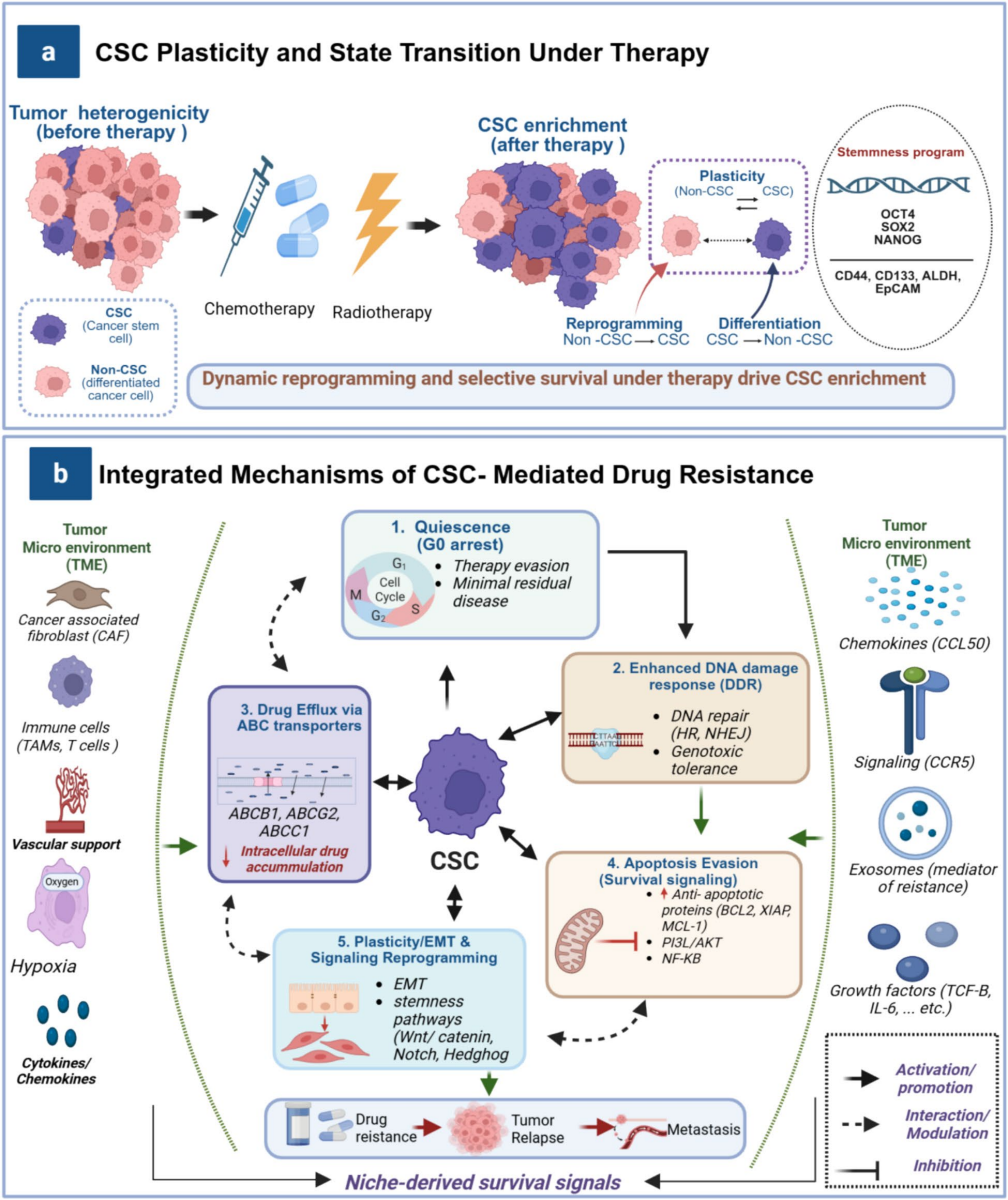
Dynamic CSC reprogramming and microenvironment-driven drug resistance. **a** Tumor heterogeneity before therapy includes CSCs and non-CSCs; therapeutic pressure induces selective survival and dynamic reprogramming, leading to CSC enrichment through bidirectional plasticity and activation of stemness programs. **b** CSCs sustain drug resistance via integrated mechanisms—quiescence, enhanced DNA damage response, drug efflux through ABC transporters, apoptosis evasion, and EMT-associated plasticity—coordinated and reinforced by tumor microenvironment signals, ultimately driving tumor relapse and metastasis

CSC-mediated drug resistance arises from the interplay between intrinsic stem cell–like characteristics and adaptive survival programs that emerge under therapeutic pressure. These mechanisms function in a coordinated, context-dependent manner that enables CSCs to survive treatment, persist over time, and ultimately repopulate tumors. Rather than acting as isolated resistance pathways, they form an integrated and dynamic network involving cellular plasticity, cell cycle regulation, enhanced DNA repair capacity, drug efflux, apoptosis evasion, and microenvironmental support (Fig. 1).

### Defining CSC properties associated with therapeutic resistance

CSCs are functionally characterized by their capacity for self-renewal, differentiation, and tumor initiation [19–23]. Within the hierarchical model of tumor organization, CSCs sustain long-term tumor growth while generating intratumoral heterogeneity, thereby enabling adaptive responses to therapeutic pressure [22–24]. Their persistence following treatment is sufficient to regenerate tumor mass and drive disease recurrence, emphasizing their central role in therapeutic failure and relapse.

Current evidence indicates that CSC identity should no longer be viewed as a fixed cellular phenotype but rather as a dynamic and context-dependent state. Stemness is regulated through coordinated interactions among epigenetic remodeling, metabolic adaptation, and microenvironmental signaling, allowing reversible transitions between stem-like and non-stem states during therapy [25–28]. This plasticity supports continual replenishment of the resistant cell population and may itself be promoted by therapeutic exposure. However, whether this phenomenon reflects true cellular reprogramming or selective expansion of pre-existing resistant clones remains unresolved [29].

Mechanistically, CSC- mediated resistance arises from the convergence of multiple processes, including EMT, deregulated signaling pathways, enhanced DNA repair, drug efflux, and apoptosis evasion [27, 30], reinforced by clonal evolution and genetic heterogeneity [4, 31, 32]. In parallel, specialized microenvironmental niches composed of stromal, vascular, and immune components provide survival cues that stabilize resistant phenotypes and maintain CSC function [32, 33]. Together, these observations support the concept that hypoxia, stemness, and therapeutic resistance operate as an interconnected functional axis.

Cellular plasticity also contributes to resistance against molecularly targeted therapies. In EGFR-mutant non-small cell lung cancer, tumor cells can undergo EMT-associated transitions toward a stem-like phenotype accompanied by reduced dependence on EGFR signaling. This phenotypic adaptation enables survival during EGFR tyrosine kinase inhibitor (TKI) treatment and illustrates how non-genetic reprogramming can promote drug tolerance independently of the canonical oncogenic pathway [34].

Another defining feature linking CSC identity to therapeutic resistance is the capacity to adopt a quiescent or slow-cycling state. This adaptive behavior allows CSCs to evade cell cycle–dependent therapies and persist as minimal residual disease [22, 35]. Maintenance of this phenotype is supported by core stemness-associated markers, including CD44, CD133, EpCAM, and ALDH, together with activation of transcriptional regulators such as OCT4, SOX2, and NANOG [36].

Nevertheless, the variability of these markers across tumor contexts highlights the limitations of rigid CSC classifications.

Overall, CSCs are best understood as dynamic and adaptive cellular states capable of surviving therapeutic stress, undergoing phenotypic reprogramming, and driving tumor repopulation. Within this framework, quiescence represents a critical functional bridge connecting stemness to therapeutic resistance.

### Cell cycle regulation and quiescence

A hallmark of CSC-mediated resistance is the ability to enter a quiescent state characterized by reversible arrest in the G0 phase, rendering these cells relatively insensitive to therapies that preferentially target actively proliferating population [37]. Importantly, quiescence is not a passive dormant condition but an actively regulated survival program that enables CSCs to tolerate cytotoxic stress while preserving long-term tumorigenic potential.

The clinical implications of quiescence are substantial. Although conventional therapies effectively eliminate rapidly dividing tumor cells, they often fail to eradicate quiescent CSC populations. These surviving cells remain capable of reinitiating tumor growth after treatment [38]. completion, creating a temporal dimension of resistance in which short-term therapeutic responses do not necessarily translate into durable disease control [37]. However, whether quiescence reflects an intrinsic stem cell property or an adaptive response induced by therapeutic stress remains incompletely understood, and resolving this question may be essential for developing effective treatment strategies.

At the molecular level, regulatory pathways controlling stemness are closely interconnected with cell cycle regulation. The Wnt/β-catenin pathway illustrates this relationship by simultaneously promoting chemoresistance and inducing cell cycle arrest, thereby supporting CSC survival under treatment condition [39].

Importantly, inhibition of Wnt signaling may reduce proliferative activity without fully eliminating tumor-initiating capacity, underscoring the limitations of strategies based solely on growth suppression. More broadly, quiescence reflects a fundamental characteristic of stem cell biology. Features such as dormancy and asymmetric division are inherently linked to enhanced stress resistance, activation of autophagy, and suppression of apoptosis [8, 39, 40]. Collectively, these mechanisms reinforce CSC survival under hostile conditions and suggest that therapeutic resistance may arise from extension of normal stem cell physiology rather than solely from acquired adaptation.

Therefore, quiescence should be considered a central component of CSC-mediated resistance, enabling temporal escape from therapy and facilitating tumor recurrence while functionally intersecting with enhanced DNA repair under genotoxic stress.

### Enhanced DNA damage response and repair capacity

Enhanced DNA damage response (DDR) represents a fundamental mechanism through which CSCs withstand genotoxic therapies, including chemotherapy and radiotherapy, both of which depend on the induction of DNA lesions to trigger cell death [4, 38]. Compared with differentiated tumor cells, CSCs possess a superior capacity to detect, repair, and tolerate DNA damage, thereby preserving genomic integrity under conditions that would otherwise compromise cell viability.

This enhanced repair capability is supported by activation of multiple DNA repair pathways, particularly homologous recombination and checkpoint signaling, which facilitate efficient resolution of DNA double-strand breaks [41]. In parallel, metabolic reprogramming provides the energetic and biosynthetic resources required to sustain repair processes, linking DDR to broader adaptive responses that support CSC survival [4]. However, an unresolved question remains whether enhanced DDR reflects an intrinsic characteristic of CSC biology or develops as an adaptive response to therapeutic stress. Clarifying this distinction may have important implications for the effectiveness of DDR-directed therapies.

Importantly, DDR should not be viewed solely as a cell-autonomous process, as accumulating evidence indicates substantial regulation by TME. Niche-derived signals have been shown to augment DNA repair activity through specific pathways, including CCL5–CCR5-mediated activation of DNA repair machinery, thereby enhancing resistance to chemotherapy [42]. These findings position the TME as an active regulator of genomic stability and suggest that targeting DDR alone may provide limited benefit unless supportive niche interactions are simultaneously disrupted [43, 44].

Functionally, enhanced DDR prevents accumulation of lethal genomic damage and limits activation of downstream apoptotic pathways, ultimately preserving CSC viability under therapeutic pressure [44]. Therapeutic approaches which target DDR including checkpoint inhibition and synthetic lethality strategies have demonstrated encouraging potential; however, functional redundancy among repair pathways and compensatory signaling mechanisms remain major obstacles to durable therapeutic responses [45].

Overall, DDR acts as a genomic safeguard that enables CSCs to survive genotoxic stress while retaining tumor-initiating capacity. Its close interaction with quiescence further supports the concept of an integrated resistance program in which reduced proliferation and enhanced repair cooperate to sustain long-term survival.

### Drug efflux via ATP-binding cassette transporters

Expression of ATP-binding cassette (ABC) transporters represents one of the classical mechanisms underlying CSC-mediated multidrug resistance. Through active drug export, these transporters reduce intracellular accumulation of chemotherapeutic agents and thereby diminish treatment efficacy [46]. Among the most extensively studied transporters, ABCB1, ABCG2, and ABCC1 are frequently enriched in CSC populations and contribute directly to therapeutic resistance across multiple tumor type [2, 27, 46].

Nevertheless, emerging evidence suggests that the biological role of ABC transporters extends beyond their function in drug efflux. Transporter activity has been closely linked to maintenance of stemness, regulation of differentiation, and modulation of survival-associated signaling pathways, supporting the view that ABC transporters operate as integrated components of the broader CSC regulatory network rather than isolated mediators of resistance [27, 46, 47]. This expanded functional role may explain the consistent association between elevated transporter expression and aggressive disease behavior as well as unfavorable clinical outcomes.

Despite strong mechanistic evidence supporting their role in resistance, therapeutic targeting of ABC transporters has achieved limited clinical success. Redundancy within the transporter family, systemic toxicity resulting from inhibition of physiological transport processes, and the persistence of alternative resistance pathways collectively reduce treatment efficacy [48]. These limitations emphasize the disconnect between mechanistic understanding and successful clinical translation and suggest that targeting transporter activity alone is unlikely to overcome CSC-driven resistance.

More recent studies have shifted attention toward the regulatory landscape governing ABC transporter function. Microenvironmental signaling, metabolic adaptation, and exosome-mediated intercellular communication have all been implicated in regulating transporter expression and activity [9]. Notably, exosome-dependent transfer of regulatory molecules may disseminate resistance-associated traits across tumor cell populations, amplifying the overall impact of ABC-mediated drug efflux [49, 50]. This emerging perspective challenges the traditional cell-intrinsic model of resistance and supports the concept of resistance propagation at the population level.

Accordingly, ABC transporters should be considered elements of a broader adaptive resistance network in which drug efflux is coordinated with complementary survival mechanisms. Their interaction with DDR pathways and apoptosis resistance highlights the need for combinatorial therapeutic approaches that target multiple levels of CSC adaptation.

### Evasion of apoptosis and pro-survival signaling

Evasion of apoptosis represents a critical endpoint of CSC-mediated therapeutic resistance, as the effectiveness of most anticancer treatments ultimately depends on activation of programmed cell death pathway [2, 8]. CSCs exhibit a pronounced anti-apoptotic phenotype that enables sustained survival under diverse stress conditions, including DNA damage, oxidative stress, and therapeutic challenge.

This resistance phenotype is mediated through coordinated remodeling of apoptotic signaling networks, including increased expression of anti-apoptotic proteins, suppression of pro-apoptotic mediators, and activation of survival-associated pathways such as PI3K/AKT and NF-κB [8]. These adaptive responses are further strengthened through clonal selection and progressive genetic evolution during treatment exposure [51]. However, an important unresolved issue is whether apoptosis resistance acts as a primary driver of CSC persistence or develops secondarily as a consequence of upstream processes such as enhanced DDR and metabolic adaptation.

Mitochondrial regulation constitutes a central component of apoptosis evasion. CSCs display altered mitochondrial dynamics, increased mitophagy, and substantial metabolic flexibility, collectively preventing activation of apoptotic cascade Furthermore, interactions between apoptosis and autophagy introduce an additional level of regulatory complexity, as cytoprotective autophagy may compensate for apoptotic signaling during therapeutic stress. This adaptive shift allows CSCs to redirect lethal stimuli into survival-promoting pathways and creates additional challenges for therapeutic intervention.

The TME further amplifies resistance by delivering survival signals and maintaining specialized protective niches that preserve CSC viability despite ongoing treatment [52]. These observations reinforce the importance of understanding apoptosis evasion within its broader microenvironmental context rather than viewing it exclusively as a cell-intrinsic phenomenon.

Taken together, apoptosis evasion functions as a convergent survival mechanism that integrates multiple upstream resistance pathways. Its central position within the CSC resistance network highlights the importance of therapeutic strategies capable of simultaneously disrupting survival signaling, metabolic adaptation, and microenvironmental support.

## The tumor microenvironment as a protective CSC niche

**Fig. 2:**
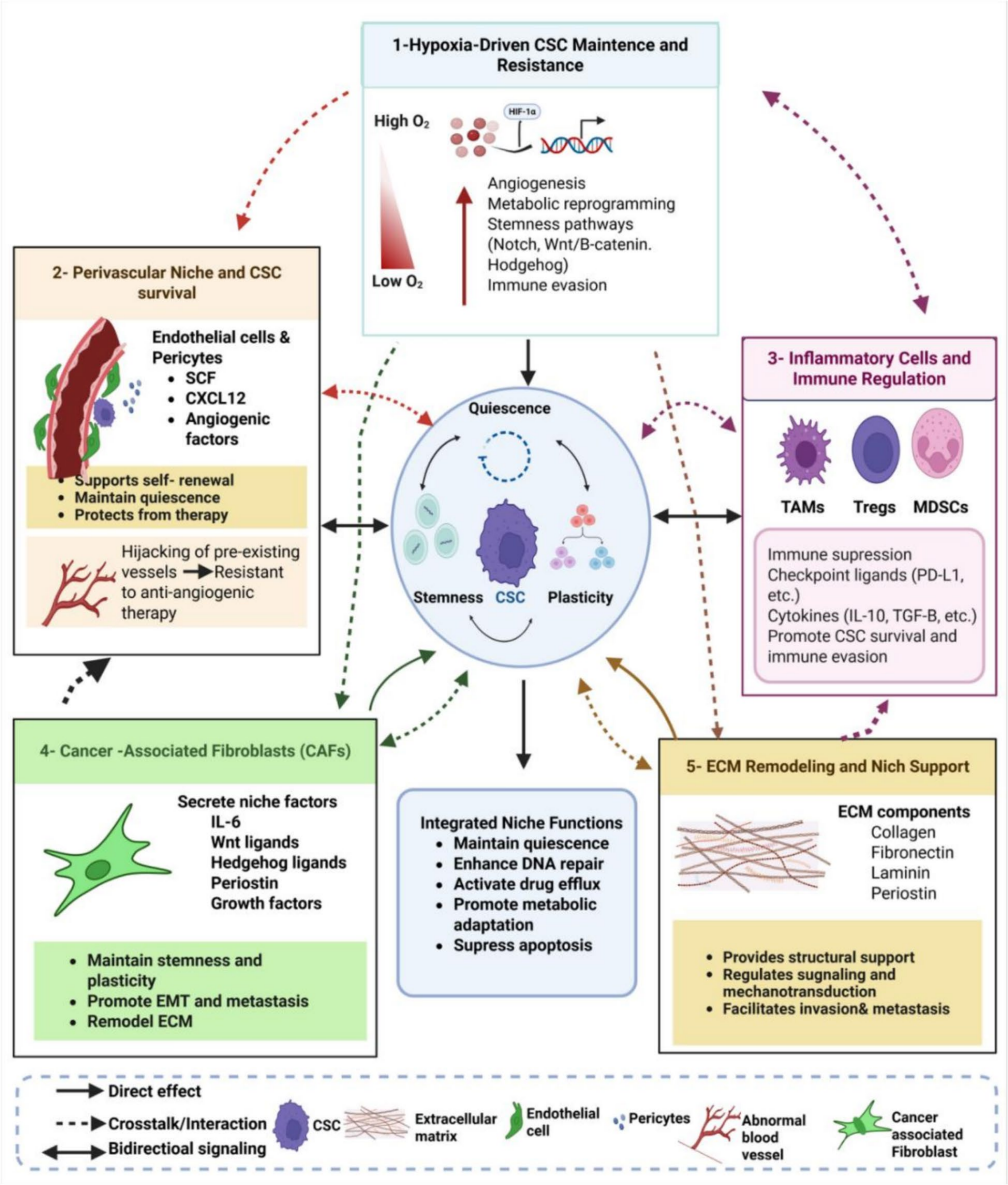
Tumor microenvironment as a dynamic CSC niche driving resistance. CSC behavior is regulated by an integrated tumor microenvironment (TME). Hypoxia activates HIF-1α signaling, promoting stemness, metabolic reprogramming, and immune evasion. The perivascular niche supports CSC self-renewal, quiescence, and protection against therapy, including through vessel co-option. Inflammatory cells, including tumor-associated macrophages (TAMs), regulatory T cells (Tregs), and myeloid-derived suppressor cells (MDSCs), establish an immunosuppressive microenvironment, whereas cancer-associated fibroblasts (CAFs) secrete factors that sustain stemness, promote epithelial–mesenchymal transition (EMT), and remodel the extracellular matrix (ECM). ECM components provide both structural and signaling support for tumor invasion and metastasis. Collectively, these niche components maintain CSC quiescence, enhance DNA repair and drug efflux, suppress apoptosis, and ultimately drive therapeutic resistance and tumor progression

The CSC niche is a dynamic and highly integrated microenvironment that regulates stemness, cellular plasticity, and therapeutic resistance. Comprising perivascular structures, inflammatory cells, cancer-associated fibroblasts (CAFs), and hypoxic regions, the niche provides both biochemical and structural support that promotes CSC survival and drives tumor evolution. Understanding how these components interact to maintain quiescence, facilitate immune evasion, and enhance metastatic potential is essential for developing therapeutic strategies that comprehensively target CSCs (Fig. 2).

### Hypoxia-driven CSC maintenance and resistance

The relationship between hypoxia and the CSC niche has evolved into a well-established mechanistic framework in which hypoxia actively regulates stemness, cellular plasticity, and therapeutic resistance. The CSC niche functions as a dynamic microenvironment that governs stem cell behavior, whereas hypoxia imposes selective pressure that favors adaptable, stem-like populations and promotes tumor evolution [53]. Accordingly, hypoxic regions serve as functional niches that enhance CSC survival by limiting drug penetration, promoting aberrant vascularization, and imposing metabolic constraints. Within these regions, CSCs preferentially localize to microenvironments characterized by oxygen gradients, extracellular matrix composition, and stromal interactions that collectively sustain stemness and cellular plasticity [54].

At the molecular level, the hypoxia-inducible factor (HIF) signaling axis plays a central role in this process. Stabilization of HIF-1α activates transcriptional programs that regulate angiogenesis, cellular metabolism, and stemness-associated signaling pathways, including Notch, Wnt/β-catenin, and Hedgehog [55–57]. In addition, HIF signaling coordinates immune evasion and therapeutic resistance through extensive interactions with the tumor microenvironment [58]. Beyond these signaling pathways, hypoxia-induced metabolic reprogramming directly contributes to CSC maintenance by linking glycolytic adaptation and redox homeostasis to stemness [57, 59]. More recently, metabolic intermediates have also been implicated in epigenetic regulation through mechanisms such as histone lactylation, although the contribution of these pathways appears to be context-dependent across different tumor types [59, 60].

Functionally, hypoxic niches promote therapeutic resistance through multiple coordinated mechanisms, including reduced drug accessibility, induction of quiescence, activation of drug efflux, enhanced DNA repair, and increased phenotypic plasticity [60–62]. These effects are further reinforced by hypoxia-driven immunosuppressive remodeling of the TME. Nevertheless, the relative contribution of these mechanisms varies according to tumor type, disease stage, and temporal changes within the microenvironment, highlighting the heterogeneous nature of hypoxia–CSC interactions [63–65].

Therapeutic strategies have consequently evolved from targeting HIF signaling alone toward combination approaches that simultaneously disrupt hypoxia-associated pathways, metabolic adaptation, and the supportive CSC niche, including emerging metabolic–epigenetic interventions [66, 67]. However, clinical translation remains limited due to pathway redundancy and adaptive resistance. In summary, the hypoxia–CSC niche should be understood as a dynamic and integrated regulatory system, in which microenvironmental signals, metabolic reprogramming, and stemness pathways converge to sustain therapeutic resistance, thereby necessitating coordinated, multi-level targeting strategies to achieve durable clinical responses [68, 69].

Overall, the hypoxia–CSC niche represents a dynamic and context-dependent system in which microenvironmental signals actively reprogram stemness and sustain therapeutic resistance. These insights highlight the need for integrated strategies that target both CSC plasticity and niche-derived support to achieve durable treatment responses.

### The perivascular niche and CSC survival

The perivascular niche has emerged as a central regulator of CSC biology, evolving from a mere vascular support structure into a complex microenvironment that orchestrates stemness, quiescence, and therapeutic resistance [70]. The spatial organization of this niche creates distinct functional zones: arteriolar regions preferentially maintain deep quiescence, whereas sinusoidal regions support proliferation and differentiation, resulting in sub-niche heterogeneity with important therapeutic implications. Within this niche, CSCs engage in dynamic bidirectional communication with endothelial cells, promoting angiogenesis while receiving paracrine signals that support self-renewal and enhance therapeutic resistance, particularly in glioblastoma [71, 72]. Importantly, the concept of the perivascular niche extends beyond neuro-oncology. For example, CD44-high alveolar type II stem cells preferentially localize adjacent to large blood vessels, where they are supported by endothelial cells and adventitial fibroblasts, resulting in increased susceptibility to KRAS-driven oncogenic transformation [73, 74].

An additional layer of complexity is introduced by vessel co-option, a process in which tumors hijack pre-existing vasculature rather than inducing new blood vessel formation. This strategy provides CSCs with a protective niche that is relatively resistant to anti-angiogenic therapies, including VEGF-targeted treatment [74–76]. Collectively, the perivascular niche integrates vascular signals (e.g., SCF, CXCL12), cellular interactions, and spatial heterogeneity to create a dynamic hub that sustains CSC survival, regulates quiescence, and promotes metastatic potential.

### Inflammatory cells and cancer-associated fibroblasts in CSC regulation

Inflammatory cells and fibroblasts are pivotal architects of the CSC niche, coordinating immune evasion, stemness maintenance, and extracellular matrix remodeling [19, 77]. CSCs are not passive entities within the tumor microenvironment; they actively remodel their surroundings, suppressing antigen presentation, upregulating immune checkpoint ligands, and recruiting immunosuppressive populations such as regulatory T cells and myeloid-derived suppressor cells [78]. Translationally, targeting tumor-initiating CSCs’ immune shields has been shown to improve immunotherapy efficacy. Inflammatory and therapeutic stress further drives CSC plasticity, enabling non-stem cancer cells to dedifferentiate into stem-like states, which contributes to metastasis through pre-metastatic niche formation guided by chemokine pathways such as CXCL12/CXCR4 [1].

Perivascular immune niches reinforce this regulatory network by organizing immune cells, stromal elements, and mesenchymal populations that maintain vascular integrity and immune homeostasis [79, 80]. CAFs complement this system by secreting IL-6, Wnt, Hedgehog ligands, and periostin, establishing reciprocal interactions with CSCs that sustain stemness and remodel the ECM [80–82]. Adventitial fibroblasts, with mesenchymal stem cell-like potential, contribute to niche construction and can differentiate into CAFs under tumor-induced remodeling, linking perivascular structure to CSC maintenance. Matricellular proteins such as periostin further exemplify the integration of ECM remodeling with immune regulation, priming the tumor microenvironment for progression [83, 84].

The convergence of perivascular structures, inflammatory cells, and fibroblasts forms a self-reinforcing triad that sustains CSC survival, therapeutic resistance, and tumor evolution. Emerging studies highlight cooperative CSC behaviors, including “altruistic” phenotypes under therapeutic stress, and underscore the importance of integrated interventions targeting CSC–niche interactions rather than CSCs alone [67]. Collectively, this evidence establishes that the CSC niche operates as a dynamically regulated ecosystem, where cellular crosstalk, biochemical signaling, and structural organization collectively determine tumor resilience, adaptability, and clinical outcomes.

## Cellular plasticity and EMT in CSC-mediated drug resistance

**Fig. 3:**
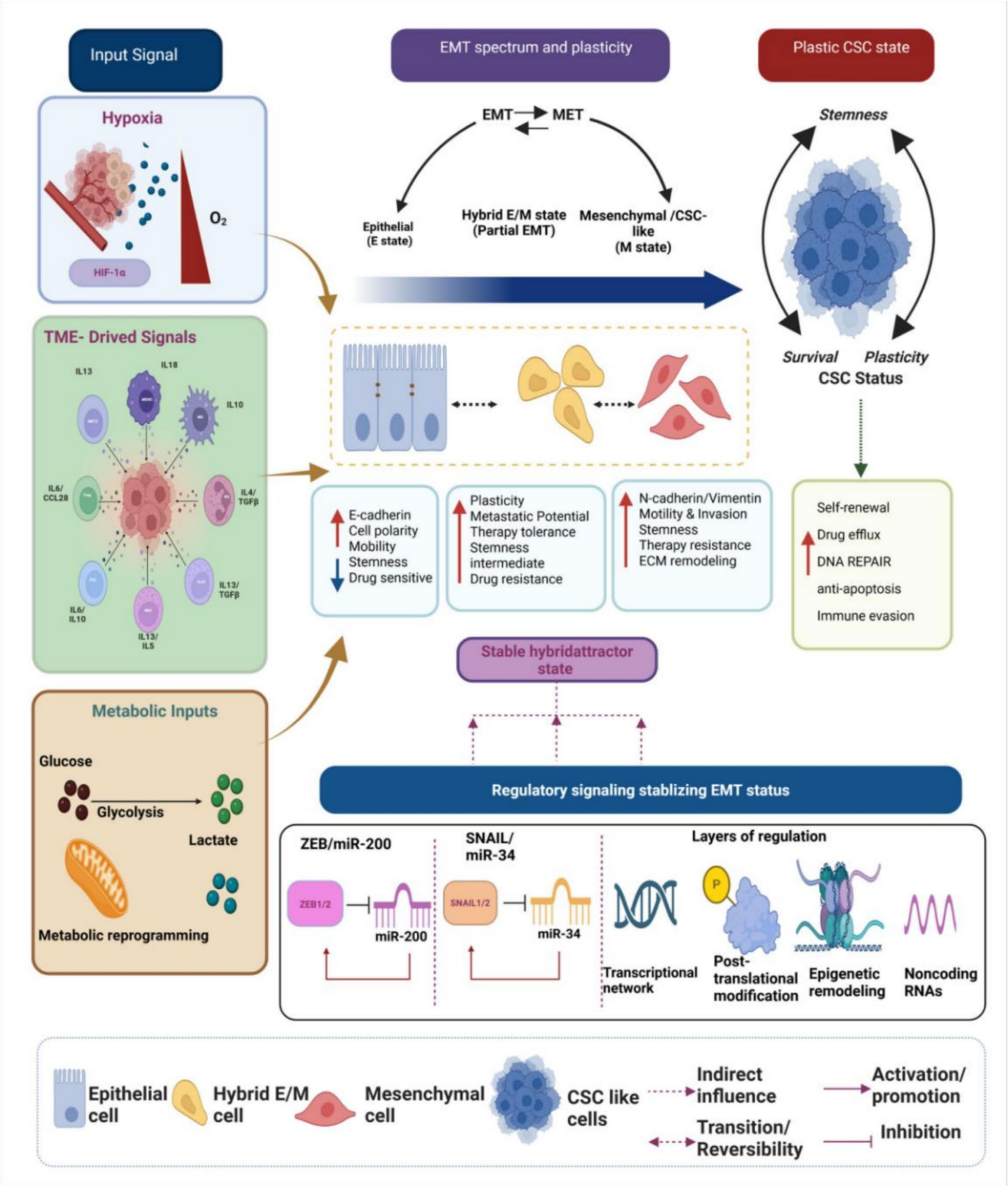
EMT plasticity and microenvironmental regulation of CSC states. CSC plasticity is driven by integrated microenvironmental and metabolic inputs. Hypoxia and TME-derived signals (cytokines, growth factors) initiate EMT, while metabolic reprogramming (e.g., glycolysis and lactate production) further supports this transition. Cells dynamically shift along the EMT spectrum from epithelial to hybrid E/M to mesenchymal states, with hybrid states representing stable, highly plastic and therapy-resistant phenotypes. This plasticity is regulated by core signaling circuits (ZEB/miR-200, SNAIL/miR-34) and multiple regulatory layers, including transcriptional, post-translational, epigenetic, and noncoding RNA mechanisms. The resulting plastic CSC state is characterized by enhanced stemness, survival, drug resistance, DNA repair, and immune evasion, highlighting EMT as a central mechanism linking plasticity to therapeutic resistance

EMT functions as a dynamic and reversible plasticity program that integrates transcriptional, metabolic, and microenvironmental signals to generate stem-like, therapy-resistant states, thereby driving CSC maintenance, immune evasion, and metastatic progression while limiting the effectiveness of single-target therapies (Fig. 3).

### EMT as a driver of stemness and resistance

EMT is a fundamental biological process that drives cancer progression and therapeutic resistance. It is regulated by key transcription factors, including SNAI1, SNAI2, ZEB1, ZEB2, and TWIST, whose aberrant expression is strongly associated with tumor aggressiveness, invasion, metastasis, and poor patient survival [85, 86]. Rather than representing a simple binary transition, EMT encompasses a continuum of intermediate cellular states. These hybrid epithelial/mesenchymal phenotypes are stable and possess enhanced stemness, tumor-initiating capacity, and metastatic potential. Computational modeling of core regulatory circuits, particularly the ZEB/miR-200 and SNAIL/miR-34 feedback loops, has demonstrated that these hybrid states function as thermodynamically stable attractors, enabling reversible phenotypic switching in response to environmental stimuli [87–91]. The concept of EMT "respecialization" further suggests that cells undergoing partial EMT acquire context-dependent phenotypes rather than progressing through a strictly linear epithelial-to-mesenchymal transition [92, 93].

EMT-associated stemness is orchestrated through coordinated transcriptional networks, post-translational modifications, epigenetic remodeling, and regulation by non-coding RNAs [94]. Among these regulatory mechanisms, the ZEB1/miR-200 feedback loop plays a pivotal role by repressing epithelial markers such as E-cadherin while simultaneously activating stemness-associated genes, including SOX2. Dominant ZEB1 expression therefore stabilizes a mesenchymal, stem-like phenotype that promotes therapeutic resistance [95, 96]. Clinically, increased EMT heterogeneity is consistently associated with poor prognosis, as greater phenotypic diversity generates therapy-resistant cellular subpopulations and facilitates Darwinian tumor evolution under therapeutic pressure [97].

EMT is also intimately linked to metabolic reprogramming. EMT-associated transcription factors regulate the expression of key metabolic enzymes, whereas metabolic intermediates reciprocally reshape the epigenetic landscape [98, 99]. For example, glycolysis-derived lactate promotes histone lactylation, thereby activating stemness-associated genes under hypoxic conditions and mechanistically linking the Warburg effect, epigenetic remodeling, and EMT-driven CSC phenotypes [57]. Additional mechanisms, including HIF-1-mediated serine biosynthesis and regulation of reactive oxygen species (ROS) homeostasis, further support CSC maintenance. Moreover, context-dependent metabolic plasticity, characterized by differential utilization of oxidative phosphorylation and glycolysis, has been observed in CD133⁺ osteosarcoma CSCs, highlighting the metabolic adaptability of these cells [59, 100].

### Cellular plasticity and phenotypic switching

The TME exerts further control over EMT plasticity. Hypoxia stabilizes HIF-1α and HIF-2α, activating EMT transcription factors (TWIST, SNAIL) and stemness pathways (Wnt/β-catenin, Notch, Hedgehog) [56]. Hypoxia integrates immune suppression, metabolic adaptation, and chemoresistance [101]. Cycling hypoxia generates more aggressive hybrid E/M states compared to sustained hypoxia, highlighting temporal oxygen dynamics.

CSCs actively remodel their niche to sustain EMT-favorable conditions. Interactions with immune cells and CAFs, together with reciprocal signaling, reinforce EMT and maintain a self-sustaining niche [77, 81, 102, 103]. Transcriptomic analyses reveal an inverse correlation between EMT-driven stemness and anti-tumor immune activity, defining a stemness-immunity axis that protects EMT-high CSCs from immune clearance [78].

Collectively, hypoxia, stemness, and therapeutic resistance constitute a self-reinforcing regulatory network interconnected through EMT, providing a mechanistic explanation for the limited efficacy of single-target therapeutic approache [104]. Tissue-specific studies further reveal the existence of altruistic CSC phenotypes, cooperative niche protection, and prolonged hypoxia-induced plasticity in head and neck, colorectal, and HepG2 tumor models [63–65].

Therapeutic strategies have progressed from single EMT transcription factor inhibition to combination approaches targeting hypoxia, niche interactions, and metabolic–epigenetic regulation [105, 106]. Liu et al. (2023) emphasize that disrupting CSC–niche interactions, rather than CSCs alone, represents the current translational frontier [67].

Overall, EMT should be regarded as a dynamic, niche-dependent program of cellular plasticity that integrates transcriptional, epigenetic, metabolic, and microenvironmental signals. Its central role in maintaining CSC stemness, promoting therapeutic resistance, and driving metastatic progression underscores the need for integrated therapeutic strategies that simultaneously target EMT regulators, CSC plasticity, and the supportive tumor microenvironment.

## Metabolic Reprogramming in Therapy-Resistant CSCs

CSCs exhibit profound metabolic plasticity that is fundamentally linked to their resistance and survival. Unlike the rigid, high-rate glycolysis of the Warburg effect seen in many differentiated cancer cells, CSCs are metabolically flexible, dynamically switching between glycolysis and oxidative phosphorylation (OXPHOS) in response to environmental and therapeutic stress.

### Metabolic plasticity: the glycolysis-OXPHOS switch

Unlike bulk tumor cells, which are often restricted to aerobic glycolysis (the Warburg phenotype), CSCs exhibit remarkable metabolic plasticity, allowing them to dynamically shift between glycolysis and OXPHOS in response to microenvironmental and therapeutic pressure [107, 108]. This metabolic flexibility ensures sustained ATP production and biosynthetic activity under adverse conditions, including hypoxia, nutrient deprivation, and chemotherapy, that would otherwise compromise the survival of metabolically less adaptable cell [107]. Mechanistically, this metabolic switch is regulated by key factors such as HIF-1α, c-Myc, and PGC-1α, which coordinate metabolic flux toward either glycolysis or mitochondrial respiration depending on cellular demands [108, 109].

Importantly, metabolic plasticity is not solely a cell-intrinsic property but is profoundly influenced by the TME. Hypoxia-associated signaling pathways, including Wnt/β-catenin and Notch, actively reprogram CSC metabolism, highlighting the pivotal role of niche-derived signals in shaping metabolic adaptation [107]. Furthermore, advances in single-cell sequencing and spatial transcriptomics have revealed considerable metabolic heterogeneity within CSC populations, demonstrating that metabolic phenotypes exist along a continuum even among stem-like cells [21, 110].

A key implication of this metabolic adaptability is that conventional anticancer therapies may unintentionally select for metabolically flexible CSC populations. Elimination of glycolysis-dependent tumor cells can enrich OXPHOS-dependent CSCs, whereas therapies targeting mitochondrial metabolism may preferentially select glycolytic CSC subpopulation [108, 110]. This reciprocal selection provides a mechanistic explanation for the limited clinical success of therapies directed against a single metabolic pathway. Beyond the glycolysis–OXPHOS axis, CSC metabolism encompasses a broader metabolic network involving alterations in the tricarboxylic acid (TCA) cycle, fatty acid oxidation (FAO), nucleotide biosynthesis, lipid metabolism, and glutathione-dependent redox pathways [111]. Notably, CSCs may display relatively lower overall metabolic activity compared to differentiated tumor cells, contributing to intrinsic resistance to therapies targeting highly active metabolic phenotypes [111].

Expanding this metabolic repertoire, CSCs also rely on alternative nutrient utilization pathways, particularly fatty acid and amino acid metabolism. FAO is frequently upregulated under metabolic stress and therapeutic pressure, providing not only ATP but also NADPH and acetyl-CoA to maintain redox homeostasis and support epigenetic regulation. Similarly, amino acid metabolism, particularly glutamine utilization, fuels TCA cycle anaplerosis, nucleotide biosynthesis, and antioxidant defense, collectively enhancing CSC survival and adaptive capacity [112].

Additional adaptive mechanisms further diversify CSC metabolism. For example, serine metabolism has emerged as a critical pathway supporting redox balance and survival during oxidative stress in pancreatic cancer models. Likewise, ovarian CSCs exploit lipid and amino acid metabolism in concert with mitochondrial remodeling to preserve stemness and promote drug resistance within hostile tumor microenvironments [112, 113].

### Redox homeostasis and oxidative stress resistance

Redox homeostasis represents a fundamental protective mechanism in CSCs. Although tumor cells generally exhibit elevated reactive oxygen species (ROS) due to increased metabolic activity, CSCs maintain relatively low intracellular ROS levels through robust antioxidant systems, preserving genomic stability, stemness, and long-term survival capacity [114–116]. This controlled redox state constitutes both a defense mechanism and a therapeutic vulnerability.

At the molecular level, CSC redox homeostasis is regulated by interconnected transcriptional networks involving NRF2, BACH1, HIF-1α, NF-κB, FOXO proteins, and AP-1 [114]. Among these, NRF2 serves as the master regulator of antioxidant responses, whereas HIF-1α integrates hypoxic signaling with metabolic and redox adaptation. More recently, BACH1 has emerged as a key regulator linking oxidative stress responses with EMT and stemness-associated program [114]. Because these pathways form highly interconnected regulatory networks, inhibition of individual components is often compensated by activation of alternative signaling pathways, limiting therapeutic efficacy.

Recent advances further recognize ROS as essential signaling molecules regulating tumor progression, immune interactions, and cellular organization [115]. However, therapeutic targeting of ROS requires precise modulation, as excessive oxidative stress may paradoxically promote genomic instability and adaptive resistance [115]. Consequently, emerging strategies focus on disrupting CSC redox balance through combinatorial approaches. For example, photoactivatable nanodisc systems induce ROS generation while simultaneously depleting glutathione, effectively impairing CSC self-renewal and promoting differentiation [116]. Similarly, oxidant-based therapies aim to overwhelm antioxidant defenses, ultimately triggering apoptosis in otherwise therapy-resistant CSC populations [117].

Importantly, metabolic plasticity and redox homeostasis are tightly interconnected. Mitochondrial function regulates not only energy production but also ROS generation and NADPH availability for antioxidant defense [110], while multi-omics analyses demonstrate that lipid metabolism, glutathione pathways, and energy production form an integrated network rather than discrete modules [111]. This systems-level perspective suggests that CSC resistance arises through coordinated metabolic and redox regulation, with mitochondria serving as a central hub that integrates these processes. Accordingly, therapeutic strategies that simultaneously target metabolic flexibility and redox buffering are more likely to overcome CSC-mediated resistance than approaches directed against either pathway alone [116].

## Immune evasion and resistance to immunotherapy in CSCs

### Mechanisms of CSC-mediated immune evasion

The interplay between CSCs and immunotherapy resistance represents one of the central challenges in modern oncology. CSCs are now recognized not merely as passive reservoirs of resistance but as dynamic regulators of immune evasion that actively remodel the TME to withstand immunotherapeutic pressure. Immune checkpoint inhibitors (ICIs), particularly those targeting PD-1/PD-L1 and CTLA-4, have significantly improved clinical outcomes in selected patient populations. However, durable responses remain limited, as both primary and acquired resistance continue to restrict their long-term efficacy [118, 119]. This resistance is driven by multiple mechanisms, including an immunosuppressive TME, metabolic reprogramming, impaired immune cell infiltration, and defective antigen presentation [120–126]. Within this context, CSCs function as key mediators of tumor persistence, relapse, and metastatic progression.

**Fig. 4:**
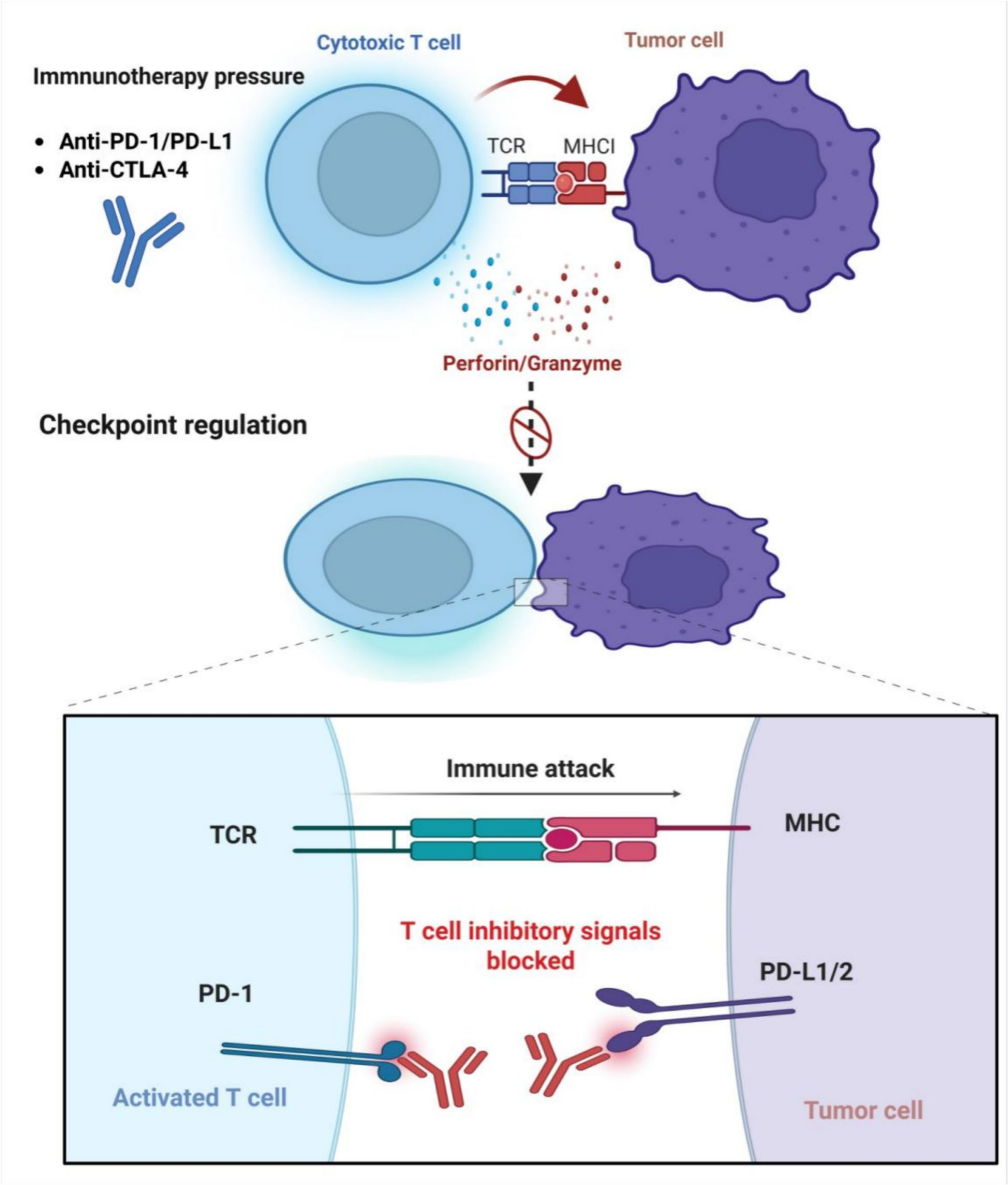
Immune checkpoint blockade and T cell–mediated anti-tumor response. Immunotherapy (anti-PD-1/PD-L1 and anti-CTLA-4) enhances cytotoxic T cell activity against tumor cells. T cell receptor (TCR) recognition of tumor antigens presented by MHC triggers immune attack through perforin and granzyme release. Under normal conditions, checkpoint interactions such as PD-1/PD-L1 inhibit T cell function and limit cytotoxicity. Immune checkpoint blockade disrupts these inhibitory signals, restoring T cell activation and enabling effective tumor cell killing

A major conceptual advance is the recognition that CSC plasticity drives immune escape. The concept of “CSC mimicry,” introduced by Saw et al., describes a therapy-induced, reversible state in which non-stem tumor cells acquire stem-like properties, thereby promoting resistance and immune evasion [18]. This challenges the traditional hierarchical model and supports a dynamic view in which stemness is inducible and context-dependent. Consistently, single-cell and spatial transcriptomic analyses reveal that CSCs exist along a continuum of phenotypic states characterized by metabolic flexibility and adaptive signaling rather than fixed identities [21]. Moreover, CSCs operate within evolving niches, and resistance is increasingly understood as a spatiotemporal process driven by CSC–TME interactions. Accordingly, targeting CSCs alone is insufficient, and disruption of the supportive niche is required for therapeutic efficacy [127] (Fig. 4).At the molecular level, CSC-mediated immune evasion is orchestrated through complex bidirectional interactions with immune cells. CSCs modulate the activity of tumor-associated macrophages (TAMs), myeloid-derived suppressor cells (MDSCs), and regulatory T cells (Tregs) through the release of cytokines, exosomes, and metabolic intermediates, thereby reinforcing an immunosuppressive tumor microenvironment [128]. A key mechanistic advance is the identification of the tumor-initiating stem cell (tSC)–driven Fads1–arachidonic acid–PGE2 axis. Guo et al. demonstrated that SOX2^high^tSCs at the tumor–stroma interface reprogram tumor-associated neutrophils through enhanced PGE2 signaling, suppressing interferon-mediated antitumor responses and promoting resistance to immunotherapy [129]. This provides direct evidence linking CSC-specific metabolic pathways to immune cell reprogramming and therapeutic failure.

Additional regulatory mechanisms further contribute to CSC-mediated immune resistance. Neurotransmitter signaling has recently emerged as a novel regulator of the TME, influencing immune checkpoint expression and immune cell function, thereby establishing a previously underappreciated neural–immune axis in immunotherapy resistance [118]. In parallel, CSCs evade T-cell-mediated cytotoxicity through activation of immune checkpoint pathways and impairment of antigen presentation [130, 131]. Collectively, these findings underscore the multifaceted nature of CSC-mediated immune escape and highlight the complexity of the interactions that support therapeutic resistance.

### Implications for immunotherapy and combination strategies

Interactions between CSCs and the immune system are highly context-dependent and vary considerably across tumor types. In colorectal cancer, CSCs modulate both innate and adaptive immune responses, establishing self-reinforcing regulatory loops that promote tumor progression and immune evasion [132]. In triple-negative breast cancer, CSCs enhance DNA damage repair while simultaneously generating protective microenvironments that reduce immune-mediated tumor clearance [133]. Similarly, in head and neck squamous cell carcinoma, the intratumoral microbiota further contributes to the establishment of immunosuppressive niches that facilitate CSC survival [134]. Together, these observations emphasize the need for tumor-specific therapeutic strategies that target CSC–immune interactions.

More broadly, CSCs should be viewed as integral components of a dynamic and reciprocal TME network. Foundational studies have demonstrated that CSCs not only depend on their surrounding microenvironment but also actively remodel it through continuous interactions with vascular, immune, and stromal compartments [33]. Early investigations further established a connection between CSC biology and immune checkpoint blockade, including telomere-targeted therapeutic approaches capable of modulating the TME to overcome therapeutic resistance [135]. Subsequent studies have strengthened the concept that CSCs function as active architects of therapeutic resistance rather than passive survivors of treatment [136, 137].

Despite substantial progress in elucidating the mechanisms of CSC-mediated immune resistance, significant translational challenges remain. Comprehensive analyses continue to highlight a persistent gap between mechanistic discoveries and clinical implementation, as most CSC-targeted immunotherapeutic strategies remain in preclinical development or early-phase clinical trials [138]. Current therapeutic approaches are increasingly converging on rational combination strategies, including inhibition of CSC-specific metabolic pathways such as the PGE2 signaling axis, disruption of CSC–immune crosstalk, and integration of advanced cellular immunotherapies, including chimeric antigen receptor (CAR)-T cells and bispecific antibodies [137, 139]. In parallel, institutional initiatives focused on acquired therapeutic resistance further emphasize the growing recognition of CSC-driven resistance as a major research priority.

Collectively, the field has evolved from descriptive observations toward a more precise mechanistic understanding of CSC-mediated immunotherapy resistance, with increasing emphasis on spatial biology and the multidimensional integration of immune, metabolic, and microenvironmental signaling networks. CSCs are now recognized as central regulators of immunotherapy resistance through their ability to dynamically interact with and reshape the tumor microenvironment. Consequently, overcoming CSC-mediated resistance will likely require integrated therapeutic strategies that simultaneously target CSC plasticity, immune evasion mechanisms, and the supportive niche that sustains long-term tumor persistence.

## Therapeutic strategies targeting CSC-mediated resistance

Given the multifaceted mechanisms underlying CSC-mediated therapeutic resistance, effective targeting of CSCs will require integrated, multi-pronged therapeutic strategies. In this section, we critically evaluate the major classes of CSC-directed therapies, highlighting their mechanisms of action, current clinical progress, and the key challenges that continue to limit their therapeutic efficacy.

### Targeting surface markers

Targeting CSC-associated surface markers has emerged as a promising therapeutic strategy because these molecules function not only as phenotypic identifiers but also as key regulators of stemness, self-renewal, and survival signaling [140, 141]. Core CSC markers, including CD133, CD44, EpCAM, and ALDH1 play active roles in regulating cell proliferation, invasion, and drug resistance, making them attractive targets for selective therapeutic intervention [142, 143].

A major challenge, however, is the substantial functional and signaling overlap between CSCs and normal stem cells, which limits the development of highly selective therapeutic approaches [144]. Consequently, current strategies focus on improving target specificity through differential antigen expression, combinatorial targeting approaches, and the identification of alternative CSC-associated antigens.

Several therapeutic modalities have been developed to exploit these surface markers. Antibody–drug conjugates (ADCs) enable receptor-mediated intracellular delivery of cytotoxic agents, thereby circumventing drug efflux-mediated resistance [145]. In addition, targeting CSC-specific glycosylation patterns—including truncated O-glycans, MUC1 variants, and gangliosides (GD2 and GD3) has expanded the repertoire of potential therapeutic targets [146]. Likewise, antibody-conjugated nanoparticle systems have demonstrated improved targeting specificity while reducing systemic toxicity [147].

Adoptive cellular therapies are also advancing rapidly. CD133-directed CAR-T cells have entered Phase II clinical trials, whereas EpCAM-targeted CAR-T therapies are currently undergoing early-stage clinical evaluation [148, 149]. Combination strategies incorporating epigenetic modulators aim to overcome CSC plasticity and prevent CSC reconstitution following treatment [150], Similarly, CAR-NK cell platforms offer improved safety profiles while maintaining antitumor activity [142, 146]. In parallel, bispecific antibodies, such as CD3–EpCAM constructs, facilitate direct T-cell engagement without the need for ex vivo manipulation [151], with multi-specific designs addressing antigen heterogeneity [152]. Additional approaches, including radioimmunotherapy (e.g., L1CAM-targeted^161^Tb) and dendritic cell–based vaccines, further extend CSC-directed strategie [151, 152].

Despite these advances, the clinical efficacy of surface marker-targeted therapies remains limited by the immunosuppressive TME, which CSCs actively establish through immune checkpoint activation and inhibitory signaling pathways [142]. The presence of a CSC-associated "immunological shield" further emphasizes the need for combination strategies that integrate CSC-targeted therapies with immune-modulating approaches [151].

Collectively, targeting CSC surface markers represents a clinically actionable strategy. However, its greatest therapeutic potential is likely to be achieved through combination approaches that simultaneously address CSC plasticity, antigen heterogeneity, and the immunosuppressive TME (Table 1).

**Table 1:** Surface marker–targeted therapeutic modalities

Therapeutic Modality	Target Markers	Mechanism of Action	Development Stage	NCT number	Ref
Antibody–Drug Conjugates (ADCs)	CD44, CD133, EpCAM, ALDH1	Antibody-mediated intracellular delivery of cytotoxic payloads	Preclinical–Phase I	NCT06265688	[153]
CD133-directed CAR-T cells	CD133	Engineered T-cell–mediated cytotoxicity	Phase II (HCC)	NCT02541370	[148]
EpCAM-directed CAR-T cells	EpCAM	Engineered T-cell targeting of EpCAM⁺ cells	Phase I	NCT03013712	[149]
CAR-T combined with epigenetic therapy	CD133/EpCAM	Dual targeting with epigenetic reprogramming	Preclinical	-	[150]
Multi-marker bispecific antibodies	Multiple CSC markers	Dual-antigen targeting to enhance selectivity	Preclinical	-	[151]
Radioimmunotherapy	L1CAM	Auger electron–mediated cytotoxicity	Preclinical	-	[152]
Glycan-targeted therapies	GD2, GD3, MUC1, O-glycans	Targeting aberrant CSC-associated glycosylation	Preclinical	-	[146]
Dendritic cell (DC)-based vaccines	CSC-associated antigens	Induction of adaptive anti-CSC immune responses	Preclinical–Phase I	-	[143]
Nanoparticle-based delivery systems	CD44, EpCAM	Antibody-guided targeted drug delivery	Preclinical	-	[143]

### Inhibition of key signaling pathways

Targeting key developmental signaling pathways represents a fundamental strategy for eliminating CSCs because their self-renewal and maintenance are largely governed by evolutionarily conserved signaling networks, particularly the Wnt/β-catenin, Hedgehog (Hh), and Notch pathways. Since these pathways are also essential for normal stem cell homeostasis, achieving effective therapeutic inhibition while minimizing toxicity remains a major challenge [140, 144]. Aberrant Wnt/β-catenin activation promotes CSC maintenance, EMT, and metastasis via nuclear β-catenin accumulation, often driven by APC mutations [154, 155]. Targeting approaches have advanced toward selectivity, with the CBP/β-catenin inhibitor PRI-724 showing synergistic effects when combined with vismodegib or erlotinib, supporting dual-pathway inhibition [156]. Proteolysis-targeting chimeras (PROTAC)-based degradation of nuclear β-catenin further refines this strategy by selectively targeting oncogenic signaling [155, 157], while multiple nodes within the pathway remain druggable [155].

Hedgehog signaling represents the most clinically advanced pathway, with Smoothened inhibitors (vismodegib, sonidegib, glasdegib) approved for specific malignancies [158, 159]. However, resistance mediated by Smoothened mutations has limited durability, prompting interest in downstream targets. The Gli inhibitor GANT61 suppresses both Wnt/β-catenin and Notch pathways, supporting multi-pathway targeting approaches [160]. Notch signaling, critical for CSC maintenance, has been targeted using γ-secretase inhibitors, although clinical application is constrained by gastrointestinal toxicity [161, 162]. Crosstalk among Wnt, Hedgehog, and Notch pathways further complicates targeting, with long non-coding RNAs (lncRNAs) acting as key regulatory mediators [163].

Additional signaling pathways also contribute significantly to CSC survival and therapeutic resistance. The Hippo/YAP-TAZ pathway promotes stemness through nuclear activation of YAP and TAZ while exhibiting extensive interaction with Wnt signaling, thereby representing another promising therapeutic target [164, 165]. STAT3 signaling is therapeutically relevant, with BBI608 (napabucasin) reaching Phase III clinical evaluation [158, 166]. The PI3K/AKT/mTOR pathway functions as a central survival network, with associated kinases providing opportunities for multi-pathway targeting [166]. Autophagy supports CSC survival under stress but can be therapeutically exploited when dysregulated [165]. Ferroptosis, an iron-dependent form of cell death, has emerged as a distinct mechanism capable of selectively eliminating CSCs via GPX4 and system Xc⁻ inhibition [165, 167].

Despite encouraging progress, a major limitation of pathway-targeted therapies is the extensive signaling crosstalk that enables compensatory pathway activation, thereby diminishing the efficacy of monotherapies [168]. LncRNAs further coordinate this network across multiple regulatory levels. Consequently, current strategies emphasize multi-pathway inhibitors, rational combinations such as PRI-724 with vismodegib, and integration with conventional chemotherapy to target both CSCs and bulk tumor cells [160, 169, 170].

Collectively, these findings indicate that durable therapeutic responses are unlikely to be achieved through inhibition of a single signaling pathway. Instead, successful CSC-targeted therapies will require integrated, multi-pathway strategies capable of overcoming signaling redundancy, compensatory crosstalk, and the remarkable adaptive resilience of CSCs (Table 2).

**Table 2:** Targeting cancer stem cell (CSC) signaling pathways—therapeutic strategies and clinical progress

Pathway	Drug/Agent	Target/Mechanism	Cancer Type(s)	Clinical Stage	NCT number	References
Hedgehog	Vismodegib	Smoothened (SMO) inhibitor	Basal cell carcinoma	FDA-approved	NCT00833417	[158, 171]
Hedgehog	Sonidegib	Smoothened (SMO) inhibitor	Basal cell carcinoma	FDA-approved	NCT01327053	[158]
Hedgehog	Glasdegib + low-dose cytarabine	SMO inhibition (combination therapy)	Acute myeloid leukemia (elderly/unfit patients)	FDA-approved	NCT01546038	[165]
Hedgehog	GANT61	GLI transcription factor inhibitor	Colorectal cancer	Preclinical	-	[160]
Wnt/β-catenin	PRI-724	CBP/β-catenin interaction inhibitor	HNSCC (tongue, hypopharynx)	Preclinical; Phase I completed	NCT01302405	[170, 172, 173]
Wnt/β-catenin	Multi-target agents	Target ligands, receptors, destruction complex, TCF/β-catenin	Colorectal, breast, HCC	Preclinical–Phase I	-	[155]
Notch	γ-Secretase inhibitors (GSIs)	Inhibit Notch receptor cleavage	Breast, pancreatic, brain	Phase I–II	NCT00106145	[161, 162]
Hippo/YAP-TAZ	YAP–TEAD inhibitors	Block YAP-mediated transcription	Multiple	Preclinical	-	[164, 165]
Autophagy	Chloroquine	Autophagy inhibition	NSCLC	Preclinical		[165]
Ferroptosis	RSL3/Erastin	GPX4 inhibition/system Xc⁻ inhibition	Multiple	Preclinical	-	[165, 167]
Multi-pathway	PRI-724 + Vismodegib	Dual Wnt/β-catenin + Hedgehog inhibition	HNSCC	Preclinical	-	[156]
Multi-pathway	PRI-724 + Erlotinib	Wnt/β-catenin + EGFR inhibition	HNSCC	Preclinical	-	[156]

### Disrupting CSC–niche interactions

CSCs reside within specialized microenvironmental niches that provide essential regulatory cues through cell–cell interactions, soluble factors, ECM components, and physical conditions such as hypoxia and tissue stiffnes [174]. These niches comprise CAFs, TAMs, mesenchymal stem cells, endothelial cells, and diverse immune cell populations that collectively sustain CSC self-renewal, plasticity, and therapeutic resistance [174]. Importantly, CSC–niche interactions are bidirectional: CSCs actively remodel the TME to reinforce immunosuppression and tumor progression, while the niche reciprocally maintains CSC stemness, forming a self-reinforcing circuit that must be therapeutically disrupted [19].

Several strategies have emerged to target CSC–niche dependencies. Anti-angiogenic approaches aim to disrupt the perivascular niche, which provides endothelial-derived signals essential for CSC maintenance; however, adaptive resistance through alternative vascularization mechanisms remains a major limitation [175]. Targeting TAMs represents another promising strategy, as these cells promote CSC stemness and immune evasion. Reprogramming TAMs from a pro-tumorigenic M2 phenotype toward an anti-tumorigenic M1 phenotype can reduce niche-derived support and suppress CSC survival [176, 177].

Extracellular vesicles, particularly exosomes, have recently emerged as major mediators of CSC–microenvironment communication. CSC-derived exosomes transfer proteins, microRNAs, long non-coding RNAs, and metabolites that promote angiogenesis, immune evasion, extracellular matrix remodeling, metastatic niche formation, and therapeutic resistance. Accordingly, therapeutic inhibition of exosome biogenesis, secretion, or uptake represents a promising strategy for disrupting CSC-dependent intercellular communication [178]

The metabolic and physicochemical characteristics of the niche also provide therapeutic opportunities. The acidic extracellular pH of the TME promotes CSC survival and therapeutic resistance, and recent strategies aim to exploit, rather than simply neutralize, this acidic environment [179]. In parallel, ECM composition and tissue stiffness regulate CSC behavior through mechanotransduction pathways, particularly Hippo/YAP-TAZ signaling, highlighting the therapeutic potential of targeting biomechanical cues to destabilize CSC identity [20]. Beyond these biochemical and mechanical influences, intracellular trafficking pathways have also emerged as important regulators of ECM dynamics. VPS35-dependent retromer recycling controls the surface localization of MT1-MMP, thereby enhancing ECM degradation and promoting metastatic dissemination, linking membrane trafficking to CSC–niche interactions [180].

Finally, immune remodeling of the niche is essential for effective CSC targeting. CSC-driven immunosuppression—mediated by immune checkpoint upregulation, recruitment of regulatory immune cells, and secretion of inhibitory cytokines—limits therapeutic efficacy [180]. The identification of an "immunological shield" surrounding tumor-initiating cells further underscores the need for combination strategies that integrate CSC-directed therapies with immune checkpoint blockade and other TME-modulating approaches [151].

Overall, disrupting the CSC niche by targeting vascular support, immune suppression, stromal signaling, and biomechanical cues represents a promising strategy to enhance therapeutic efficacy and reduce tumor recurrence (Table 3).

**Table 3:** Niche-targeting strategies

Niche Component	Strategy	Mechanism	Stage	Ref
Perivascular niche	Anti-angiogenic agents	Disrupt vascular support for CSCs	Clinical (multiple agents)	[175, 181]
TAMs	M2 → M1 reprogramming	Shift macrophage polarity to anti-tumorigenic	Preclinical–Phase I	[142]
Acidic microenvironment	pH modulation/exploitation	Target acid-adaptive CSC survival	Preclinical	[179]
ECM/mechanical stiffness	Mechanotransduction disruption	Destabilize YAP/TAZ-mediated stemness	Preclinical	[20, 181]
Immune microenvironment	Checkpoint inhibitors + CSC targeting	Disarm immunological shield of CSCs	Clinical combinations	[151]
CAFs/stromal cells	Stromal depletion/reprogramming	Remove stromal support for CSC maintenance	Preclinical	[174]
Hypoxic niche	HIF inhibitors	Block hypoxia-driven stemness programs	Preclinical–Phase I	[5]

### Induction of CSC differentiation

Differentiation-based therapy aims to eliminate the stem-like phenotype of CSCs by targeting the signaling pathways that maintain their undifferentiated state, including Wnt/β-catenin, Hedgehog, Notch, and Hippo/YAP-TAZ [164, 165]. Inhibition of these pathways can relieve the differentiation block and promote maturation toward less tumorigenic phenotypes.

Hedgehog pathway inhibitors, such as the FDA-approved Smoothened inhibitors vismodegib and sonidegib, have demonstrated the ability to induce differentiation in basal cell carcinoma. However, resistance mediated by Smoothened mutations limits their long-term therapeutic efficacy [158]. Targeting downstream components, such as Gli transcription factors using GANT61, simultaneously disrupts Wnt/β-catenin and Notch signaling in colorectal CSCs, providing a strategy to overcome resistance associated with single-pathway inhibition [160].

Similarly, inhibition of Notch signaling using γ-secretase inhibitors promotes cell-fate commitment toward differentiation. However, their clinical application remains limited by dose-dependent gastrointestinal toxicity resulting from effects on normal intestinal stem cells [161, 162]. Activation of bone morphogenetic protein (BMP) signaling represents another differentiation-inducing strategy, particularly in glioblastoma, where it promotes terminal differentiation into non-tumorigenic cell lineages.

Despite these advances, CSC plasticity remains a major challenge because differentiated tumor cells can reacquire stem-like characteristics, thereby replenishing the CSC pool. Consequently, differentiation therapy is unlikely to be effective as a standalone treatment and is increasingly being incorporated into combination therapeutic strategies. The differentiation–cytotoxic paradigm, in which differentiation-inducing agents sensitize CSCs while conventional therapies eliminate their differentiated progeny, has shown considerable promise [162, 169]. In parallel, epigenetic modulators are being investigated to stabilize the differentiated phenotype and prevent dedifferentiation-mediated tumor relapse [150, 182]. A clinically important consideration is differentiation syndrome, a potentially severe inflammatory complication associated with rapid tumor cell maturation. Originally described in acute promyelocytic leukemia, differentiation syndrome has also been reported during treatment with IDH inhibitors, highlighting the need for careful clinical monitoring when differentiation-based therapies are employed.

### Targeting metabolic vulnerabilities

CSCs exhibit a distinct metabolic phenotype characterized by remarkable plasticity, enabling dynamic switching between glycolysis, oxidative phosphorylation (OXPHOS), and alternative energy sources such as glutamine and fatty acids in response to microenvironmental stress [21, 109]. This metabolic adaptability supports CSC survival under hypoxia, nutrient deprivation, and therapeutic pressure and is further reinforced by metabolic crosstalk with stromal and immune cells, creating a supportive niche that promotes therapeutic resistance [21, 152]. Importantly, CSC metabolic states are highly heterogeneous and context-dependent, making single-pathway metabolic targeting insufficient because compensatory metabolic switching readily occurs [183, 184]. Moreover, whether metabolic reprogramming is a driver or a consequence of CSC stemness remains a matter of ongoing debate, highlighting a major challenge in developing precise metabolic therapies [185].

Mitochondrial metabolism represents a key therapeutic vulnerability, as many CSCs rely predominantly on OXPHOS for energy production [110]. Agents such as metformin, which inhibit mitochondrial complex I, have therefore been extensively investigated for CSC targeting, particularly in combination with chemotherapy or naturally derived bioactive compounds [127]. Notably, these metabolic interventions are closely interconnected with epigenetic regulation and immune modulation, as metabolic reprogramming directly influences stemness maintenance and EMT.

To overcome CSC metabolic flexibility, dual inhibition of glycolysis and OXPHOS has emerged as a rational strategy to prevent adaptive metabolic escape [186]. In parallel, lipid metabolism, particularly fatty acid oxidation (FAO) which supports CSC survival and self-renewal under metabolic stress, with FAO inhibitors demonstrating encouraging preclinical efficacy across multiple tumor types. Amino acid metabolism also contributes substantially to CSC maintenance, as glutamine and serine metabolic pathways support biosynthesis, redox homeostasis, and epigenetic regulation. Consequently, therapeutic targeting of these metabolic pathways has entered early clinical evaluation [187].

Redox homeostasis represents another critical metabolic vulnerability of CSCs. Their elevated antioxidant capacity enables resistance to oxidative stress, whereas disruption of redox balance through glutathione depletion or thioredoxin inhibition can sensitize CSCs to anticancer therapy [186]. Furthermore, ferroptosis—an iron-dependent form of regulated cell death driven by lipid peroxidation—has recently emerged as a promising strategy for selectively eliminating CSCs through inhibition of glutathione peroxidase 4 (GPX4) [188].

Collectively, these findings indicate that successful metabolic targeting of CSCs will require integrated therapeutic strategies capable of overcoming metabolic plasticity, intratumoral heterogeneity, and the close interplay between metabolic, epigenetic, and microenvironmental regulatory networks (Table 4).

**Table 4:** Emerging multi-pathway and metabolic targeting strategies in CSC therapy

Pathway/Strategy	Drug/Approach	Target/Mechanism	Cancer Type(s)	Clinical Stage	NCT number	References
Wnt/Hh/Notch crosstalk	lncRNA-targeting approaches (ASOs, small molecules)	Disrupt lncRNA-mediated pathway crosstalk	Multiple	Preclinical	—	[163]
Wnt/β-catenin + PI3K	PRI-724 + HS-173	Dual inhibition of β-catenin and PI3K	HNSCC	Preclinical	—	[170]
Multiple CSC pathways	Broad-spectrum kinase inhibitors	Target RTKs, JAK/STAT, PI3K/AKT/mTOR	Multiple (HCC emphasis)	Preclinical–Phase III	Multiple	[166]
Metabolic/mitochondrial	Metformin	Mitochondrial complex I inhibition	Multiple	Phase I–II (combination)	NCT01697566 (breast cancer) or NCT01579812 (pancreatic cancer)	[170]
Dual metabolic targeting	Glycolysis + OXPHOS inhibition	Blocks metabolic plasticity	Multiple	Preclinical	—	[189]
Redox metabolism	BSO, Auranofin	Glutathione depletion and ROS induction	Multiple	Preclinical–Phase I	NCT01737502	[188]
Multi-pathway integration	Small-molecule pipelines	Combined inhibition of Wnt, Notch, Hh, STAT3, Hippo; activation of Hippo/ferroptosis	Multiple	Preclinical–Phase III	Multiple	[165]
Epigenetic + signaling	HDAC inhibitors (vorinostat, panobinostat) + pathway inhibitors	Epigenetic reprogramming + signaling blockade	Multiple	Phase I–II	NCT00091559	[172, 190]

### Epigenetic targeting strategies

CSCs are sustained by aberrant epigenetic programs including DNA methylation, histone modifications, chromatin remodeling, and non-coding RNA networks that maintain self-renewal while suppressing differentiation [172, 182]. Unlike genetic alterations, epigenetic modifications are reversible, providing a strong rationale for therapeutic intervention. Recent studies further demonstrate that epigenetic regulation operates in close coordination with both the tumor microenvironment and cellular metabolism. Metabolites such as α-ketoglutarate, acetyl-CoA, NAD⁺, and the oncometabolite 2-hydroxyglutarate directly influence the activity of epigenetic enzymes, thereby linking metabolic reprogramming to CSC maintenance [189, 191]. Advances in single-cell omics and CRISPR-based epigenetic editing have further revealed the complexity and dynamic plasticity of CSC epigenomes, supporting the development of increasingly precise epigenetic therapeutic strategies [182].

Several classes of epigenetic drugs have been developed to target CSCs. Histone deacetylase (HDAC) inhibitors, the most clinically advanced class, promote chromatin relaxation and reactivate silenced tumor suppressor and differentiation-associated genes, thereby reducing CSC stemness and overcoming chemoresistance [172, 190]. DNA methyltransferase (DNMT) inhibitors, including azacitidine and decitabine, similarly restore normal gene expression programs by reversing aberrant DNA hypermethylation and facilitating CSC differentiation [172]. Targeting histone methylation through EZH2 inhibitors, such as tazemetostat, disrupts repressive chromatin states that maintain CSC identity [21]. In addition, non-coding RNAs, particularly long non-coding RNAs (lncRNAs), function as central regulators of signaling crosstalk in CSCs, making them attractive therapeutic targets capable of modulating multiple resistance pathways simultaneously [182].

Beyond DNA and histone modifications, RNA epigenetic regulation has recently emerged as an important determinant of CSC plasticity. N6-methyladenosine (m6A), the most abundant internal RNA modification, regulates the stability, translation, and splicing of transcripts controlling CSC self-renewal, epithelial–mesenchymal transition (EMT), metastasis, and therapeutic resistance. Dysregulation of m6A writers (METTL3/METTL14), erasers (FTO and ALKBH5), and reader proteins contributes to CSC maintenance, highlighting RNA epigenetic enzymes as promising therapeutic targets that may complement conventional DNA methyltransferase and histone-modifying inhibitors [192]

Epigenetic therapies demonstrate the greatest therapeutic potential when incorporated into combination treatment strategies. Integration with CAR-T cell therapy can enhance tumor antigen expression and sensitize CSCs to immune-mediated killing [172]. Combination with chemotherapy improves drug sensitivity by modulating resistance pathways [172]. Pairing with differentiation therapy helps stabilize the differentiated state and prevent phenotypic reversion [140]. Additionally, the close interplay between metabolism and epigenetics supports combined metabolic–epigenetic targeting strategies [189, 191].

Emerging technologies continue to expand the potential of this field. CRISPR-dCas9–based epigenetic editing enables locus-specific regulation of gene expression with substantially greater precision than conventional epigenetic drugs, although efficient in vivo delivery remains a significant challenge [172]. In parallel, artificial intelligence-driven analyses and liquid biopsy–based epigenetic biomarkers are advancing precision oncology by enabling identification of patient-specific epigenetic vulnerabilities and facilitating real-time monitoring of therapeutic responses [182].

Collectively, epigenetic therapies represent a highly adaptable strategy for targeting CSCs, particularly when integrated with complementary therapeutic modalities to overcome CSC plasticity and multiple resistance mechanisms (Table 5).

**Table 5:** Epigenetic therapeutic strategies

Target	Drug Class/Agent	Mechanism in CSCs	Cancer Type	Clinical Stage	NCT Number	Ref
HDACs (pan)	Vorinostat (SAHA)	Hyperacetylation → reactivate TSGs + differentiation genes; overcome chemoresistance	CTCL; solid tumors (investigational)	FDA-approved (CTCL)	NCT00091559	[172]
HDACs (pan)	Panobinostat	Broad chromatin remodeling; CSC sensitization	Multiple myeloma; solid tumors	FDA-approved (MM)	NCT01023308	[172, 190]
HDACs (isoform-selective)	Next-gen selective HDACi	Improved therapeutic window via isoform selectivity	Multiple	Preclinical–Phase I	Depends on the specific inhibitor	[190]
DNMTs	Azacitidine, decitabine	Demethylate silenced TSG/differentiation gene promoters	MDS, AML; solid tumors (investigational)	FDA-approved (MDS/AML)	NCT00071799	[77, 150]
EZH2 (H3K27me3)	Tazemetostat	Remove repressive H3K27me3 marks → promote differentiation	Epithelioid sarcoma, FL; CSCs broadly	FDA-approved (sarcoma, FL)	NCT00260832	[152]
LSD1 (H3K4me)	Iadademstat	Modulate H3K4 methylation marks governing stemness	AML, SCLC	Phase I–II	NCT02601950	[152]
BET/BRD4	JQ1, OTX015	Disrupt super-enhancer transcription; downregulate stemness TFs (MYC, SOX2)	Multiple	Phase I–II	NCT02875223	[152]
Epigenome + CAR-T	HDACi/DNMTi + CD133/EpCAM CAR-T	Upregulate surface antigens + destabilize CSC state + direct killing	Multiple	Preclinical	NCT01713582	[150]
Epigenome + chemotherapy	Epidrug → chemotherapy (sequential)	Re-express drug sensitivity genes; overcome efflux pump upregulation	Multiple	Phase I–II	—	[140, 172]
LncRNAs	ASOs, small molecules	Disrupt lncRNA-mediated Wnt/Hh/Notch crosstalk	Multiple	Preclinical	—	[163]
Locus-specific editing	CRISPR-dCas9 + epigenetic effectors	Precision silencing of stemness genes or activation of differentiation genes	Multiple	Preclinical (technology)	Depends on drug combination	[182]
Epigenetic biomarkers	Liquid biopsy cfDNA methylation	Non-invasive monitoring of CSC burden and treatment response	Multiple	Translational	—	[182]

## Conclusion and outlook

The recognition of CSCs as central drivers of therapeutic resistance, tumor relapse, and metastasis has fundamentally reshaped modern concepts of cancer biology and treatment. Over the past two decades, the field has progressed from the descriptive identification of CSCs to a mechanistic understanding of their remarkable resilience. CSC-mediated resistance is now recognized not as a single process but as an emergent property of a highly integrated and adaptive biological system. This system is sustained through the convergence of intrinsic cellular programs including quiescence, enhanced DNA damage repair, ABC transporter activity, and apoptosis evasion—with dynamic and reciprocal interactions within the TME.

One of the most important conceptual advances has been the recognition of CSC plasticity as a fundamental determinant of therapeutic failure. CSCs possess the capacity to undergo bidirectional phenotypic transitions, including EMT and MET, dynamically reprogram their metabolism through glycolysis–OXPHOS switching, and alter surface marker expression in response to therapeutic pressure. This remarkable adaptability enables CSCs to evade single-agent therapies and sustain long-term tumor persistence. Consequently, the traditional hierarchical model of CSC biology has evolved toward a more dynamic paradigm in which stemness is considered a transient, inducible, and context-dependent cellular state. Simultaneously, extensive crosstalk among major signaling pathways—including Wnt, Hedgehog, Notch, Hippo, and PI3K/AKT together with interactions involving hypoxia, CAFs, TAMs, and other TME components- establishes highly redundant survival networks that substantially reduce the effectiveness of monotherapies.

Despite significant progress, several important challenges continue to impede clinical translation. These include the absence of specific and universally applicable CSC markers, an incomplete understanding of the molecular mechanisms governing CSC plasticity, limited knowledge of CSC–immune interactions, and the considerable metabolic heterogeneity that exists within CSC populations.

Looking ahead, meaningful advances in CSC-directed therapy will depend on the rational development of integrated, multi-level therapeutic strategies. These approaches should incorporate dynamic combination therapies, target CSC plasticity together with niche-dependent survival mechanisms, exploit context-specific vulnerabilities, and integrate CSC-directed interventions with immunomodulatory therapies. Furthermore, advances in single-cell sequencing, spatial transcriptomics, CRISPR-based functional screening, and artificial intelligence-driven drug discovery are expected to accelerate the identification of more effective therapeutic targets. Ultimately, overcoming CSC-mediated therapeutic resistance will require a systems-level treatment framework capable of addressing the dynamic, adaptive, and heterogeneous nature of CSCs and the tumor ecosystems in which they reside.

## Data Availability

Not applicable.

## Abbreviations

ABC: ATP-binding cassette
ADC: Antibody–drug conjugate
ALDH: Aldehyde dehydrogenase
AP-1: Activator protein 1
BACH1: BTB and CNC homology 1
BMP: Bone morphogenetic protein
CAF: Cancer-associated fibroblast
CAR-T: Chimeric antigen receptor T cell
CAR-NK: Chimeric antigen receptor natural killer cell
CCR5: C-C chemokine receptor type 5
CD: Cluster of differentiation
CSC: Cancer stem cell
CTLA-4: Cytotoxic T-lymphocyte–associated protein 4
CXCL12: C-X-C motif chemokine ligand 12
CXCR4: C-X-C motif chemokine receptor 4
DDR: DNA damage response
DNMT: DNA methyltransferase
ECM: Extracellular matrix
EMT: Epithelial–mesenchymal transition
EpCAM: Epithelial cell adhesion molecule
FOXO: Forkhead box O
GPX4: Glutathione peroxidase 4
HDAC: Histone deacetylase
Hh: Hedgehog
HIF: Hypoxia-inducible factor
HIF-1α: Hypoxia-inducible factor 1 alpha
ICIs: Immune checkpoint inhibitors
IL-6: Interleukin-6
lncRNA: Long non-coding RNA
MAPK: Mitogen-activated protein kinase
MDSC: Myeloid-derived suppressor cell
NF-κB: Nuclear factor kappa B
NRF2: Nuclear factor erythroid 2–related factor 2
OXPHOS: Oxidative phosphorylation
PD-1: Programmed cell death protein 1
PD-L1: Programmed death-ligand 1
PGE2: Prostaglandin E2
PI3K/AKT: Phosphoinositide 3-kinase/protein kinase B
PGC-1α: Peroxisome proliferator-activated receptor gamma coactivator 1 alpha
PROTAC: Proteolysis-targeting chimera
ROS: Reactive oxygen species
SCF: Stem cell factor
SOX2: SRY-box transcription factor 2
STAT3: Signal transducer and activator of transcription 3
TAM: Tumor-associated macrophage
TCA: Tricarboxylic acid
TGF-β: Transforming growth factor beta
TME: Tumor microenvironment
tSC: Tumor-initiating stem cell
VEGF: Vascular endothelial growth factor
Wnt: Wingless/Integrated signaling pathway
YAP/TAZ: Yes-associated protein/Transcriptional coactivator with PDZ-binding motif
